# Hollow Hafnium Oxide (HfO_2_) Fibers: Using an Effective Combination of Sol–Gel, Electrospinning, and Thermal Degradation Pathway

**DOI:** 10.1021/acs.langmuir.3c03484

**Published:** 2024-02-20

**Authors:** Meng-Ru Huang, Yi-Fan Chen, Bhaskarchand Gautam, Yen-Shen Hsu, Jhih-Hao Ho, Hsun-Hao Hsu, Jiun-Tai Chen

**Affiliations:** † Department of Applied Chemistry, National Yang Ming Chiao Tung University, Taiwan 300093; ‡ Center for Emergent Functional Matter Science, National Yang Ming Chiao Tung University, Taiwan 300093

## Abstract

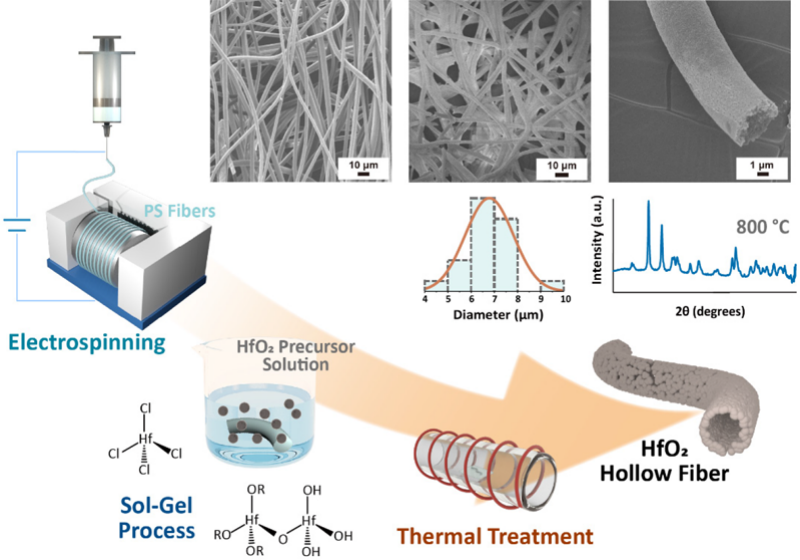

In recent years, hafnium oxide (HfO_2_) has gained increasing interest because of its high dielectric constant, excellent thermal stability, and high band gap. Although HfO_2_ bulk and film materials have been prepared and well-studied, HfO_2_ fibers, especially hollow fibers, have been less investigated. In this study, we present a facile preparation method for HfO_2_ hollow fibers through a unique integration of the sol–gel process and electrospinning technique. Initially, polystyrene (PS) fibers are fabricated by using electrospinning, followed by dipping in a HfO_2_ precursor solution, resulting in HfO_2_-coated PS fibers. Subsequent thermal treatment at 800 °C ensures the selective pyrolysis of the PS fibers and complete condensation of the HfO_2_ precursors, forming HfO_2_ hollow fibers. Scanning electron microscopy (SEM) characterizations reveal HfO_2_ hollow fibers with rough surfaces and diminished diameters, a transformation attributed to the removal of the PS fibers and the condensation of the HfO_2_ precursors. Our study also delves into the influence of precursor solution molar ratios, showcasing the ability to achieve smaller HfO_2_ fiber diameters with reduced precursor quantities. Validation of the material composition is achieved through thermogravimetric analysis (TGA) and energy-dispersive spectroscopy (EDS) mapping. Additionally, X-ray diffraction (XRD) analysis provides insights into the crystallinity of the HfO_2_ hollow fibers, highlighting a higher crystallinity in fibers annealed at 800 °C compared with those treated at 400 °C. Notably, the HfO_2_ hollow fibers demonstrate a water contact angle (WCA) of 38.70 ± 5.24°, underscoring the transformation from hydrophobic to hydrophilic properties after the removal of the PS fibers. Looking forward, this work paves the way for extensive research on the surface properties and potential applications of HfO_2_ hollow fibers in areas such as filtration, energy storage, and memory devices.

## Introduction

Hafnium oxide (HfO_2_) has been widely used in metal–oxide–semiconductor devices,^1^ optoelectronics,^2^ and microelectronics^3^ because of its high-*k* dielectric constant,^4^ excellent mechanical property,^5^ thermal stability,^6^ and wide band gap energy.^7^ The HfO_2_ materials with special morphologies have been applied to different fields. For example, Qiu et al. have applied the coating method by nano-HfO_2_ fibers on a W–Re alloy, showing a thermal shock resistance with high melting point (above 3000 K).^8^ Besides, Kahro et al. have reported a HfO_2_ film grown by atomic layer deposition (ALD), revealing the applications of resistive switching media.^9^ These applications are based on the physicochemical properties of HfO_2_ materials and the high surface areas of the nanostructures.

Other than HfO_2_ bulk materials, HfO_2_ materials with special morphologies have been prepared using various approaches, such as atomic layer deposition,^10−12^ electrochemical anodization,^13−15^ thermal oxidation,^6,16,17^ and the sol–gel process.^18−20^ For example, atomic layer deposition has been used to generate ultrathin HfO_2_ layers, which can further be used in Li–S batteries,^21^ gas sensing,^22^ and high-performance field emitters.^2^ Besides, Su et al. used electrospinning of sol–gel solutions to generate HfO_2_ nanobelts.^23^ Similarly, Xu et al. used the electrospinning method to produce HfO_2_ nanofibers.^24^ Moreover, Berger et al. used an electrochemical process to produce HfO_2_ nanotubes from pure Hf foils.^25^ Despite these works, it is still challenging to develop economical ways to fabricate HfO_2_ materials with special morphologies, especially HfO_2_ hollow fibers, considering their high specific surface areas.

To this end, in this work, we present a facile and versatile strategy to prepare HfO_2_ hollow fibers using a combination of sol–gel and electrospinning methods. First, polystyrene (PS) fibers are formed by the electrospinning technique. Subsequently, the electrospun PS fibers are coated with a HfO_2_ precursor solution, forming the HfO_2_-coated PS fibers after drying. By thermal treatment at a high temperature (800 °C), the HfO_2_ precursors condense and the PS fibers are selectively pyrolyzed, resulting in the formation of HfO_2_ hollow fibers. From the scanning electron microscopy (SEM) characterizations, the HfO_2_ hollow fibers show relatively rougher surfaces and reduced diameters, caused by the condensation of the HfO_2_ precursors and the disappearance of the PS fibers. We also investigate the effect of the molar ratios of the precursor solutions, presenting that HfO_2_ fibers with smaller diameters can be obtained using fewer amounts of the precursors. We also confirm the compositions of the prepared materials with thermogravimetric analysis (TGA) and energy-dispersive spectroscopy (EDS) mapping. Moreover, the X-ray diffraction (XRD) analysis studies display that the crystallinities of the HfO_2_ hollow fibers annealed at 800 °C are higher than those annealed at 400 °C. Furthermore, a water contact angle (WCA) of 38.70 ± 5.24° is obtained for the HfO_2_ hollow fibers, caused by the nature of the hydrophilic HfO_2_ and the removal of the hydrophobic PS fibers. Compared with other studies,^24^ our works demonstrate a versatile strategy to fabricate not only HfO_2_ fibers but also HfO_2_ hollow fibers. In addition, the combination of our polymer and sol–gel method requires lower processing temperature (400 or 800 °C) to obtain crystalline HfO_2_ materials. We believe that HfO_2_ hollow fibers may have great application potentials in various fields, such as filtration, energy storage, and memory devices.

## Results and Discussion

**Figure 1:**
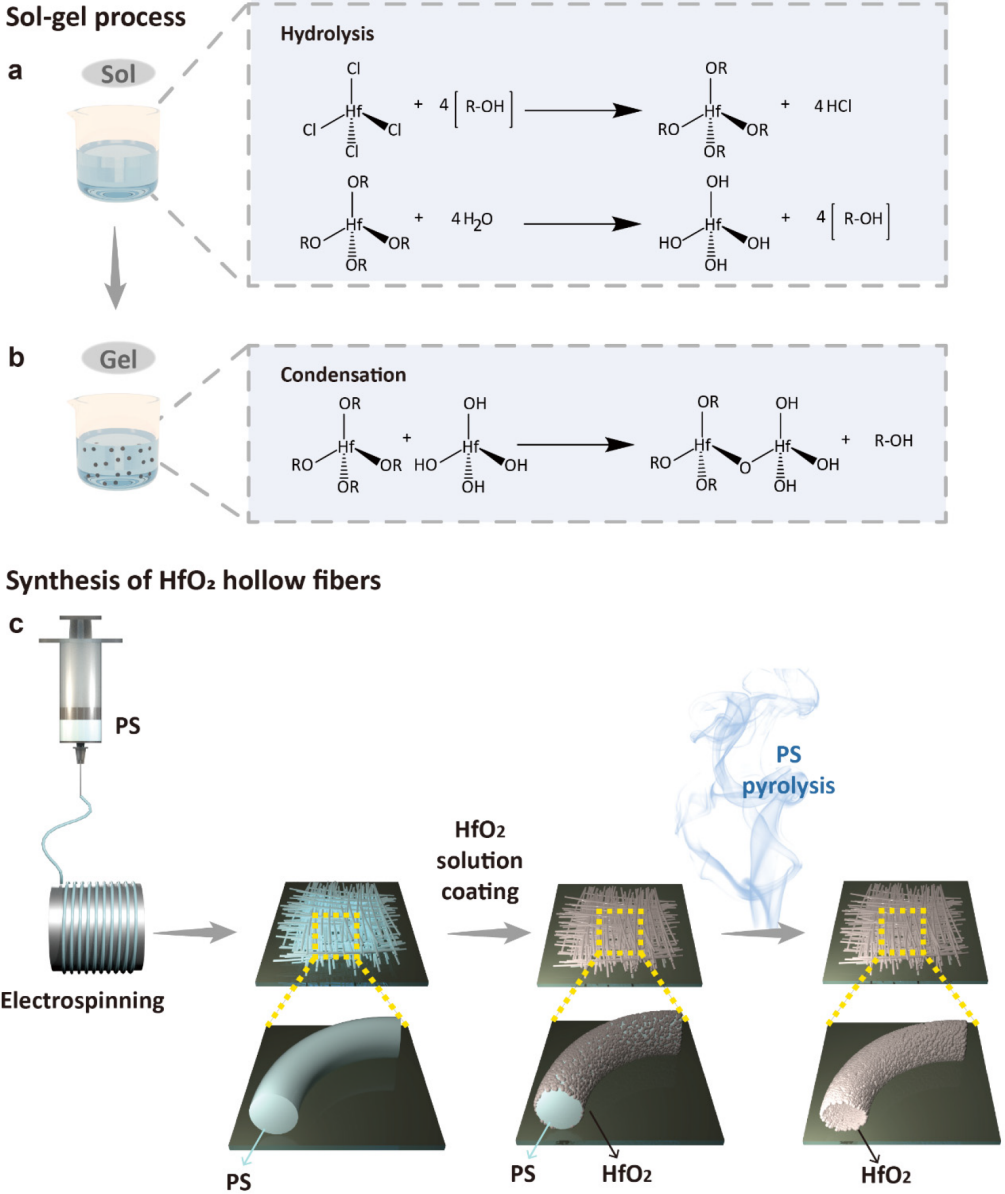
(a, b) Sol–gel process and reaction schemes of HfO_2_ formation. (c) Schematic illustrations depicting electrospinning, HfO_2_ coating, and pyrolysis to produce the HfO_2_ hollow fibers.

To prepare metal oxide nanomaterials at relatively lower temperatures, the sol–gel method is an ideal alternative to simplifying the process. Figure 1a,b shows the sol–gel process and reaction schemes of HfO_2_ formation. Hafnium(IV) chloride (HfCl_4_) is first dissolved in an organic solvent (propylene glycol methyl ether, PGME) to form HfO_2_ precursors. The hafnium atom in the complex is highly electrophilic, which tends to be attacked by oxygen and forms a hafnium alkoxide. Later, the hafnium alkoxide complex in the aqueous solution undergoes hydrolysis by replacing the alkyl group with the hydroxy group of water molecules (Figure 1a). Subsequently, the alkylated and hydrolyzed hafnium goes through polycondensation to produce a thick hafnium complex gel (Figure 1b). To understand the impact of reaction times and solvents during the gelation process, we also conducted dynamic light scattering (DLS) experiments on the sol–gel time, as shown in Figure S1.

Figure 1c shows the schematic illustrations depicting electrospinning, HfO_2_ coating, and pyrolysis to produce HfO_2_ hollow fibers. At first, a PS solution (20% w/w) in DMF is electrospun into PS fibers. Subsequently, the electrospun PS fibers are coated with a HfO_2_ precursor solution using the dip-coating method to form the HfO_2_-coated PS fibers. For the annealing temperature, we chose 800 °C according to previous studies for obtaining HfO_2_ materials with crystalline structures.^18,26^ After the thermal treatment at a high temperature (800 °C), the PS fibers are selectively pyrolyzed, and the HfO_2_ precursors condense, resulting in the formation of HfO_2_ hollow fibers. The sol–gel process is also conducted on silicon wafers to observe the relationships between the material properties and reactions. Upon the thermal treatments, the thicknesses of the HfO_2_ layers are reduced with the increases of temperature, revealing the evaporation of PGME solvents and the condensation of the HfO_2_ layers, as shown in Figure S2.

**Figure 2:**
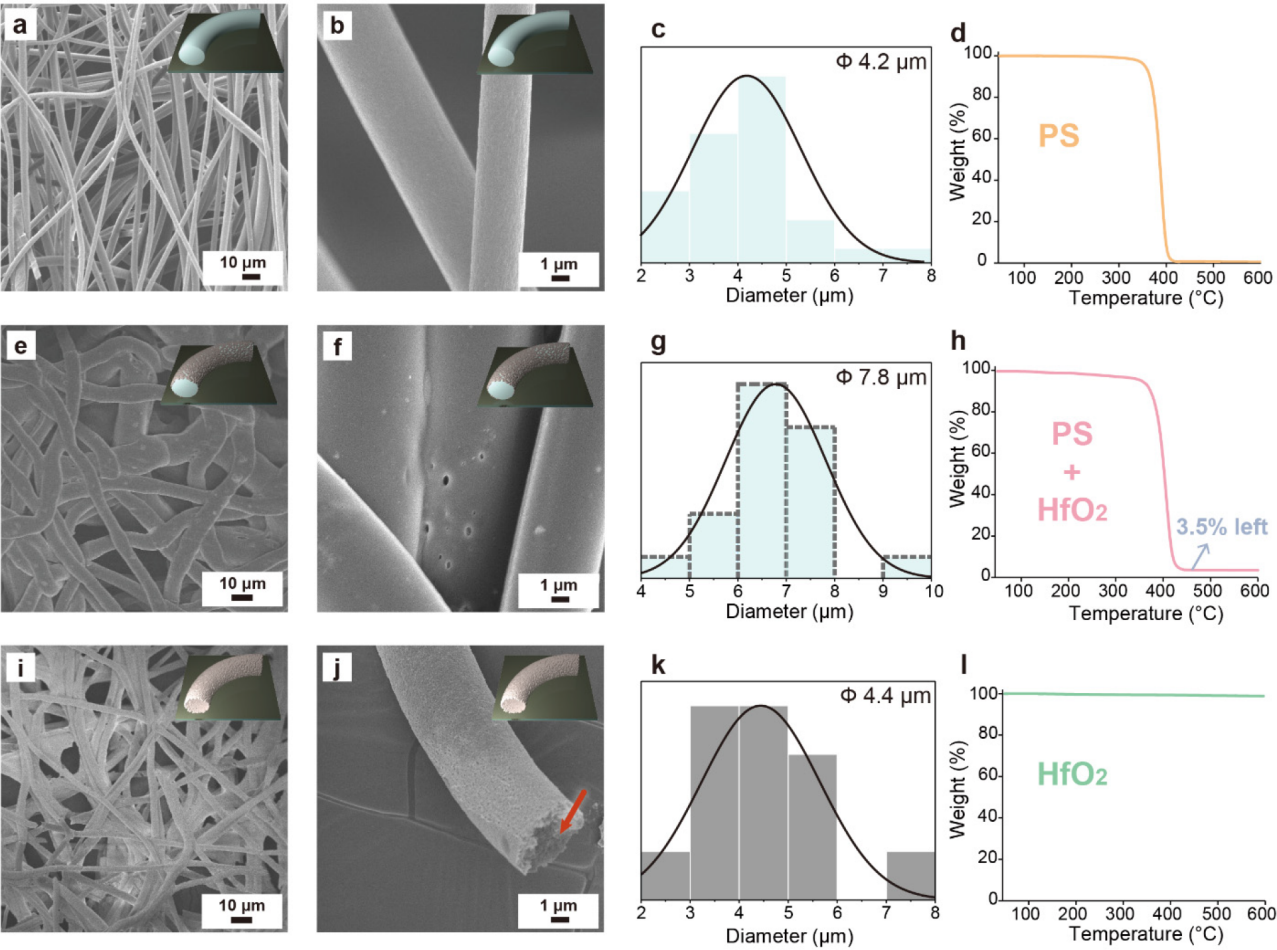
SEM images of PS fibers (a, b), HfO_2_ coated fibers (e, f), and HfO_2_ hollow fibers (i, j) with their corresponding fiber diameter distributions (c, g, k) and TGA graphs (d, h, l).

The morphologies of the PS fibers, HfO_2_ coated fibers, and HfO_2_ hollow fibers are examined by scanning electron microscopy (SEM), as shown in Figure 2. The SEM images of the electrospun PS fibers are shown in Figure 2a,b. The surfaces of the PS fibers are relatively smooth, with an average diameter of 4.2 μm, as displayed in Figure 2c. The thermogravimetric analysis (TGA) of the PS fibers demonstrates that the PS polymers remain stable at temperatures below ∼300 °C, as shown in Figure 2d. The 5 wt % weight loss of the PS polymers occurs at ∼350 °C, and complete degradation appears at ∼430 °C. The SEM images of the HfO_2_ precursor-coated PS fibers are shown in Figure 2e,f. The diameters of the fibers increased to 7.8 μm, as displayed in Figure 2g. The TGA curve of the HfO_2_ precursor-coated PS fibers demonstrates that the weights decrease significantly at ∼400 °C, mostly attributed to the loss of PS fibers. It should be noted that ∼3.5 wt % mass is still left even when the temperature is increased to 600 °C, indicating the presence of HfO_2_ even at high temperatures.

For the HfO_2_ hollow fibers, relatively rougher surfaces are observed in the SEM images (Figure 2i,j). The opening of a HfO_2_ hollow fiber is indicated by a red arrow. The diameters of the hollow fibers decrease to 4.4 μm compared with those of the HfO_2_ precursor-coated PS fibers, as displayed in Figure 2k. The reduction of the fiber diameter can be attributed to the disappearance of the PS fibers as well as the condensation of the HfO_2_ precursors. The TGA measurement of the HfO_2_ hollow fibers demonstrates that no weight loss is observed even when the temperature is increased to 600 °C, confirming the thermal stability of the hollow HfO_2_ fibers.

**Figure 3:**
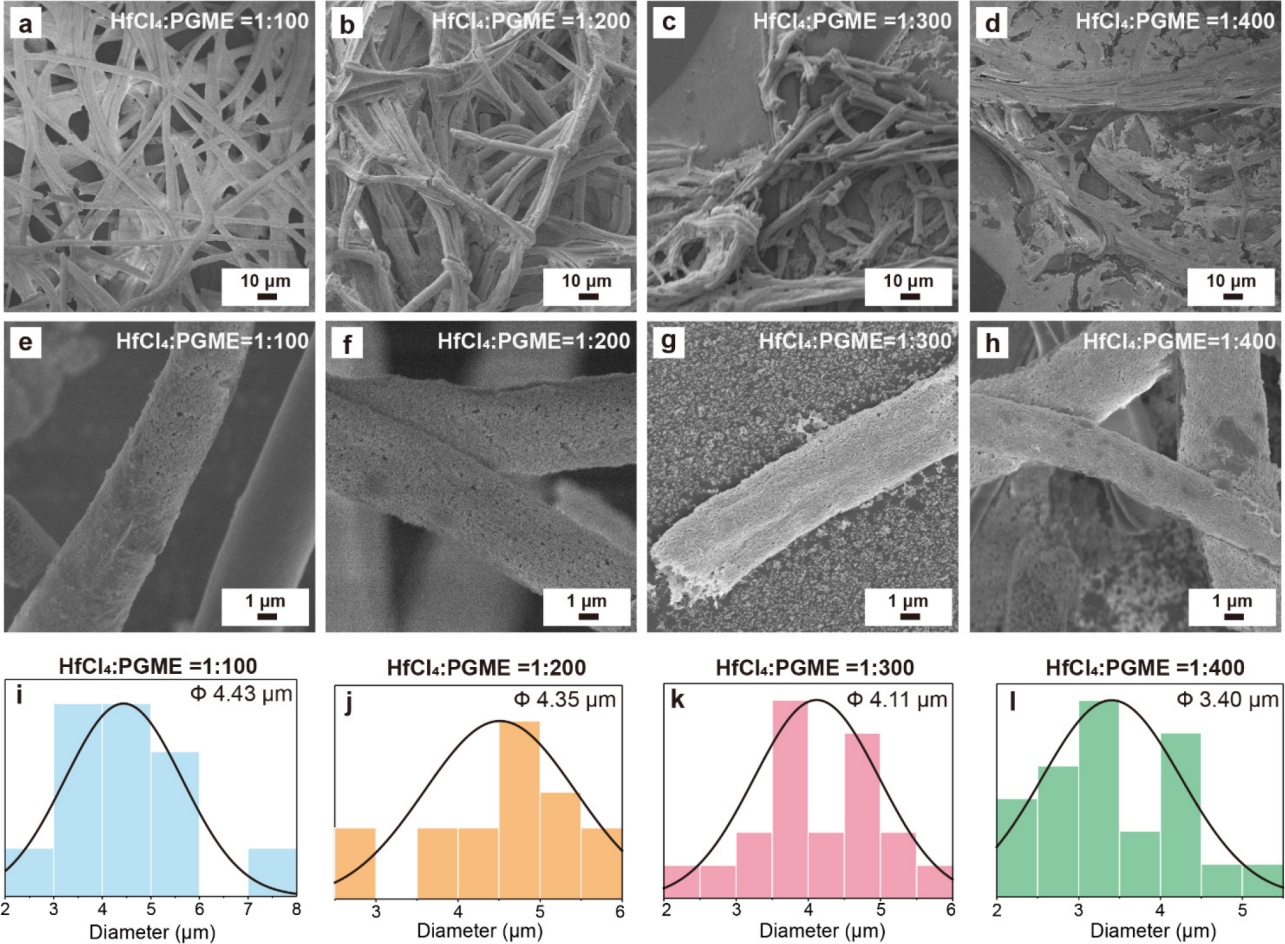
SEM images of HfO_2_ hollow fibers with different molar ratios of HfCl_4_ to PGME: (a, e) 1:100, (b, f) 1:200, (c, g) 1:300, and (d, h) 1:400. (i–l) Corresponding diameter distributions of the fibers.

We also investigated the effect of the PGME molar ratios. Figure 3 shows the SEM images of the HfO_2_ hollow fibers prepared with different HfCl_4_ to PGME molar ratios (1:100, 1:200, 1:300, and 1:400). It can be seen that the HfO_2_ hollow fibers can all be successfully fabricated. The thicknesses of HfO_2_ layers on silicon wafers are also observed via a profilometer, as shown in Figure S3. Because the total amounts of HfCl_4_ to PGME are fixed, the more amounts of PGME as the solvents are added in the sol–gel precursor, the lower the HfO_2_ thicknesses are observed. Moreover, as the amount of PGME increases, more collapsed and ribbon-like structure can be observed. Besides, for the molar ratios of 1:100, 1:200, 1:300, and 1:400, the average diameters of the HfO_2_ hollow fibers are recorded as 4.43, 4.35, 4.11, and 3.40 μm, respectively. The smaller diameters are caused by the fewer amounts of the precursors. For further characterizations, the molar ratio of HfCl_4_:PGME = 1:100 is mainly chosen to prepare HfO_2_ hollow fibers because of their relatively better surface morphologies and larger diameters. Previous works have reported the importance of the solvent in determining gelation reaction rates, indicating the viscosity and hydration effects in the precursor solutions.^27^ The solvation effect in different sol–gel precursor solvents has been tested, as displayed in Figure S4.

**Figure 4:**
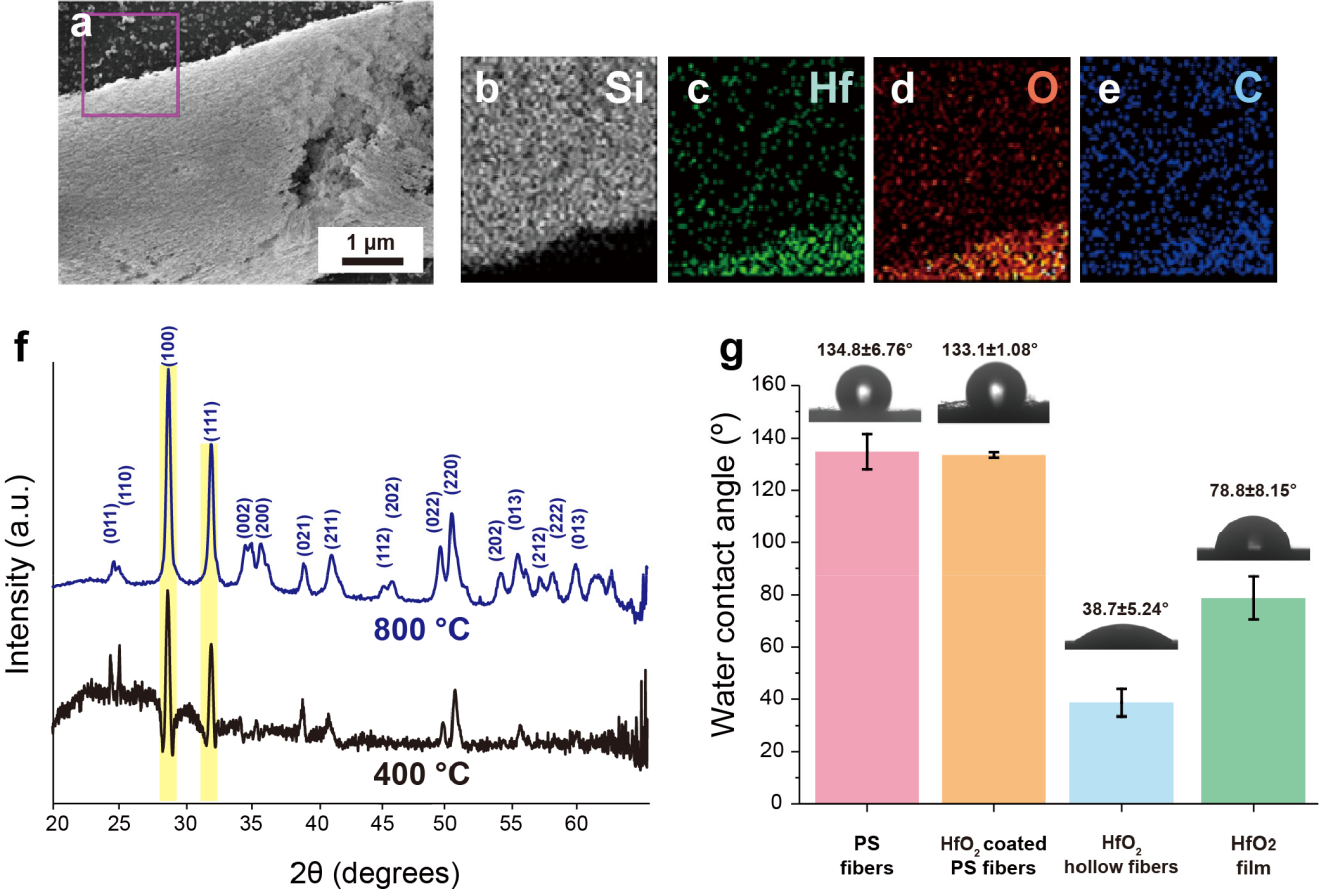
(a) SEM image of a HfO_2_ hollow fiber with corresponding EDS images: (b) Si, (c) Hf, (d) O, and (e) C. (f) XRD spectra of HfO_2_ hollow fibers after annealing at 400 (black) and 800 °C (blue). (g) Water contact angles (WCA) of PS fibers, HfO_2_ coated PS fibers, HfO_2_ hollow fibers, and HfO_2_ films coated on silicon substrates.

The HfO_2_ hollow fibers are also characterized by energy-dispersive spectroscopy (EDS) mapping, X-ray diffraction (XRD), and water contact angle (WCA) measurements. Figure 4a–e shows the SEM image of an HfO_2_ hollow fiber with corresponding EDS images. Figure 4b displays the signal of the Si element, which is mainly from the silicon wafer under the fibers, while the HfO_2_ hollow fiber does not show the Si signal. Figures 4c and 4d show the signals of the Hf and O elements, respectively, that appear on the locations of the fibers, demonstrating the formation of HfO_2_. Figure 4e is the mapping image of the C element; the C signals that appeared indicate the presence of the residual carbon after the high-temperature pyrolysis of the PS fibers. The X-ray photoelectron spectroscopy (XPS) experiments are also conducted to measure the surface compositions of the fibers, as shown in Figure S5. After pyrolysis of the PS fibers, the signals of C 1s attributed to PS decrease; in contrast, the signals of O 1s, Hf 4p, Hf 4d, and Hf 4f increase, indicating the reduction of the polymer and the formation of HfO_2_.

Furthermore, HfO_2_ hollow fibers are investigated by X-ray diffraction (XRD) measurements. Figure 4f shows the XRD curves of HfO_2_ hollow fibers using a molar ratio of [HfCl_4_]:[PGME] = 1:100 and annealing at 400 °C (black) and 800 °C (blue). The peaks at ∼29° and ∼32° can be attributed to the (100) and (111) planes of monoclinic HfO_2_. For the HfO_2_ hollow fibers annealed at 800 °C, however, more crystalline peaks with higher intensities can be observed than those annealed at 400 °C. The results show that annealing at higher temperatures can improve the crystalline structures of the HfO_2_.

In addition, the surface properties of the samples are also evaluated. Figure 4g shows the water contact angle (WCA) of PS fibers, HfO_2_ coated PS fibers, HfO_2_ hollow fibers, and HfO_2_ films coated on silicon substrate at room temperature with a water drop size of 10 μL. For PS fibers, the WCA is 134.8 ± 6.76°, which is reasonable considering the hydrophobic nature and fiber structures.^28,29^ Interestingly, the WCA of the HfO_2_ coated PS fibers is still high (133.1 ± 1.08°), probably due to the partial coverage of the HfO_2_ coating on the PS fibers. As the lotus effect, these structures are likely to be in the Cassie–Baxter state, suggesting surface-roughness-enhanced hydrophobicity. After thermal treatments, the pyrolysis of the PS fibers is conducted, maintaining the hollow HfO_2_ fiber structures. The HfO_2_ hollow fibers exhibit a much lower WCA (38.70 ± 5.24°), caused by the removal of the hydrophobic PS fibers and the nature of the hydrophilic HfO_2_. The WCAs of the HfO_2_ hollow fibers are lower than those of HfO_2_ films coated on silicon substrates As shown in Figures 2i,j and 3a–h, the HfO_2_ layers of the hollow fibers have a granular texture with some pores (∼10 nm) on the surfaces. Those structures could cause the hollow HfO_2_ fibers to become porous and water-absorbing materials, related to the Wenzel state. Compared with the pure HfO_2_ films with hydrophilic surfaces caused by OH groups, the hollow HfO_2_ fibers with high porosity present a lower WCA because of water absorption by the rough surfaces of the HfO_2_ hollow fibers.

## Conclusions

In this work, we successfully synthesize HfO_2_ hollow fibers using a combination of sol–gel and electrospinning methods. After the formation of the PS fibers using electrospinning, HfO_2_ precursor solution are coated on the electrospun PS fibers, forming the HfO_2_-coated PS fibers. By thermal treatment at a high temperature (800 °C), the PS fibers are selectively pyrolyzed, and the HfO_2_ precursors condense, resulting in the formation of HfO_2_ hollow fibers. The morphologies of the prepared materials are examined by SEM; HfO_2_ hollow fibers with relatively rougher surfaces and reduced diameters are observed, attributed to the disappearance of the PS fibers and the condensation of the HfO_2_ precursors. The effect of the molar ratios of the precursor solutions is also investigated, demonstrating that HfO_2_ fibers with smaller diameters can be obtained using the fewer amounts of the precursors. TGA and EDS mapping are also applied to confirm the compositions of the prepared materials.

Furthermore, the XRD results indicate that the crystallinities of the HfO_2_ hollow fibers annealed at 800 °C are higher than those annealed at 400 °C. In addition, the HfO_2_ hollow fibers exhibit a WCA of 38.70 ± 5.24°, caused by the removal of the hydrophobic PS fibers and the nature of the hydrophilic HfO_2_. In the future, we will further study the surface properties and applications of the HfO_2_ hollow fibers such as filtration, energy storage, and memory devices.

## Experimental Section

### Materials

Polystyrene (PS) (*M*_w_: 192000 g/mol) was purchased from Sigma-Aldrich. Nitric acid (HNO_3_) (60%) was obtained from J. T. Baker. Dimethylformamide (DMF) and hafnium(IV) chloride (HfCl_4_) (98%) were purchased from Echo Chemical. 1-Propanol (99%), ethanol (99%), and 1-methoxy-2-propanol (PGME, 99.5%) were purchased from Uni-Onward. The chemicals were used without further purification.

### Preparation of Polystyrene (PS) Fibers through Electrospinning

The electrospinning process was based on our previous work with some modifications.^29,30^ A PS (20 wt %) solution in DMF was prepared in a 20 mL glass vial. This solution was added to a 5 mL syringe, which was further attached to a capillary nozzle having an inner diameter of 0.41 mm. The syringe containing the polymer solution was connected to the pump. A 10 kV voltage was provided by using a power supply (SIMCO). A grounded rotating aluminum drum (rotating speed: 800 rpm) was used to collect the electrospun fibers. The working distance between the nozzle and the collector was kept constant at 15 cm. The spinning duration was ∼3 h for forming the pieces of fabrics. After the electrospinning process, PS fibers were dried at room temperature and stored for analysis and further processing.

### Preparation of Hafnium Oxide Sol–Gel Precursors

To prepare hafnium oxide (HfO_2_) sol–gel precursors, 1 mol of hafnium tetrachloride (HfCl_4_) was dissolved in a 20 mL glass vial containing propylene glycol methyl ether (PGME) (100 mol) at room temperature with constant stirring. HNO_3_ acid (4 mol) was then added dropwise to the solution and stirred until a clear solution mixture was observed. The solution was further heated at 100 °C for 2 h using a hot plate (Corning PC-420D) with a stirring speed of 100 rpm. After 2 h, the prepared gel solution was stored at 4 °C for further experiment. PGME with other ratios (200, 300, and 400 mol) were also prepared using the same protocol to study the solvent concentration effect on the fibers.

### Preparation of Hollow Hafnium Oxide Fibers

The PS fiber mat (1 cm × 1 cm) was dipped into a hafnium oxide sol–gel solution for 1 min at room temperature. After coating, the fibers were washed using DI water three times (1 min for each time) to ensure the removal of the nonbounded gel solution. The washed fibers were dried by using a vacuum pump. Finally, the dried fibers were kept in a heating furnace at 800 °C using a heating rate of 1 °C/min to degrade the PS polymer from the fibers, producing HfO_2_ hollow fibers.

### Characterization

A scanning electron microscope (SEM, JEOL JSM-7401F) and energy-dispersive X-ray spectroscopy (EDS, Hitachi SU-8010) were used to measure the fiber morphologies and the elementals present in the fiber interior by using an acceleration voltage of 5 kV. A thermogravimetric analysis (TGA) was used to determine the thermal stability and degradation of the PS fibers. The crystallinities of the HfO_2_ fibers were observed using X-ray diffraction (XRD) with beamline 13A1 of the National Synchrotron Radiation Research Center (NSRRC) located at Hsinchu, Taiwan. The wettabilities of the fiber surfaces were determined using a digidrop contact angle meter (goniometer, FTA 125) with deionized water droplets with sizes of 10 μL. For the annealing process, a heating furnace (tube furnace, Linberg/Blue, HTF55347C) was used with a heating rate of 1 °C/min.

## Supporting Information Available

DLS experiments for different storage times with different sol–gel solvents and different storage temperatures; HfO_2_ thicknesses at different molar ratios and in different temperatures; top-view SEM images and fiber diameter distributions of the hollow HfO_2_ fibers prepared with different solvents; XPS data of the HfO_2_ coated PS fibers and HfO_2_ hollow fibers (PDF)
