# Supplementary material for: Hollow Hafnium Oxide (HfO2) Fibers: Using an Effective Combination of Sol–Gel, Electrospinning, and Thermal Degradation Pathway
Source: Langmuir. 2024 Feb 20;40(9):4732–8. doi: 10.1021/acs.langmuir.3c03484 (PMC10919084; doi:10.1021/acs.langmuir.3c03484)
Supplement: Supplementary file 1 — la3c03484_si_001.pdf [file la3c03484_si_001.pdf]

Supporting Information

Hollow Hafnium Oxide (HfO_2) Fibers: Using an Effective Combination of Sol-Gel, Electrospinning, and Thermal Degradation Pathway

Meng-Ru Huang,[1,†] Yi-Fan Chen, [1,†] Bhaskarchand Gautam,[1] Yen-Shen Hsu,[1] Jhih-Hao Ho,[1] Hsun-Hao Hsu,[1] and Jiun-Tai Chen[1,2*]

[1]Department of Applied Chemistry, National Yang Ming Chiao Tung University, Taiwan 300093

[2]Center for Emergent Functional Matter Science, National Yang Ming Chiao Tung University, Taiwan 300093

[†]Meng-Ru Huang and Yi-Fan Chen contributed equally to this work.

TABLE OF CONTENTS

1. DLS experiments for different storage times with different sol-gel solvents S-2
2. HfO_2 thicknesses in different temperatures S-3
3. HfO_2 thicknesses at different molar ratios S-4
4. Hollow HfO_2 fibers prepared with different solvents S-5
5. XPS data of the HfO_2 coated PS fibers and HfO_2 hollow fibers S-6

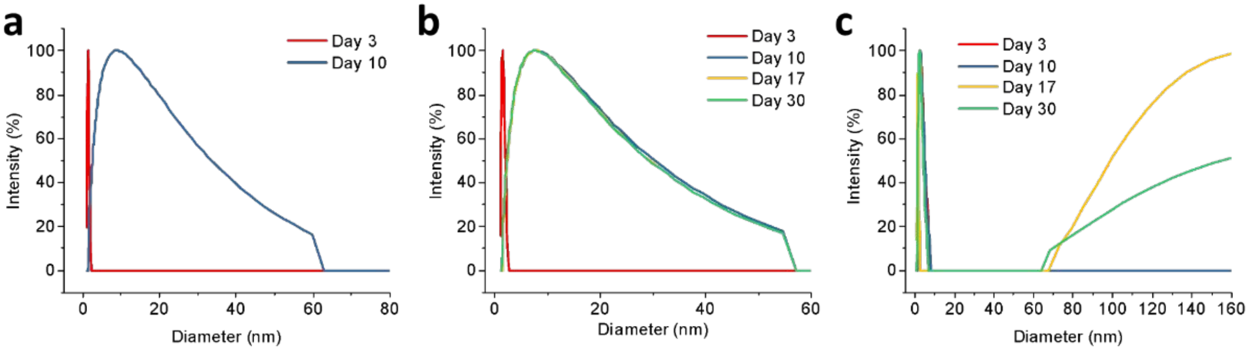

Figure S1. DLS experiments for different storage times with different sol-gel solvents: (a) ethanol at room temperature, (b) ethanol kept at 5 °C, and (c) PGME at room temperature.

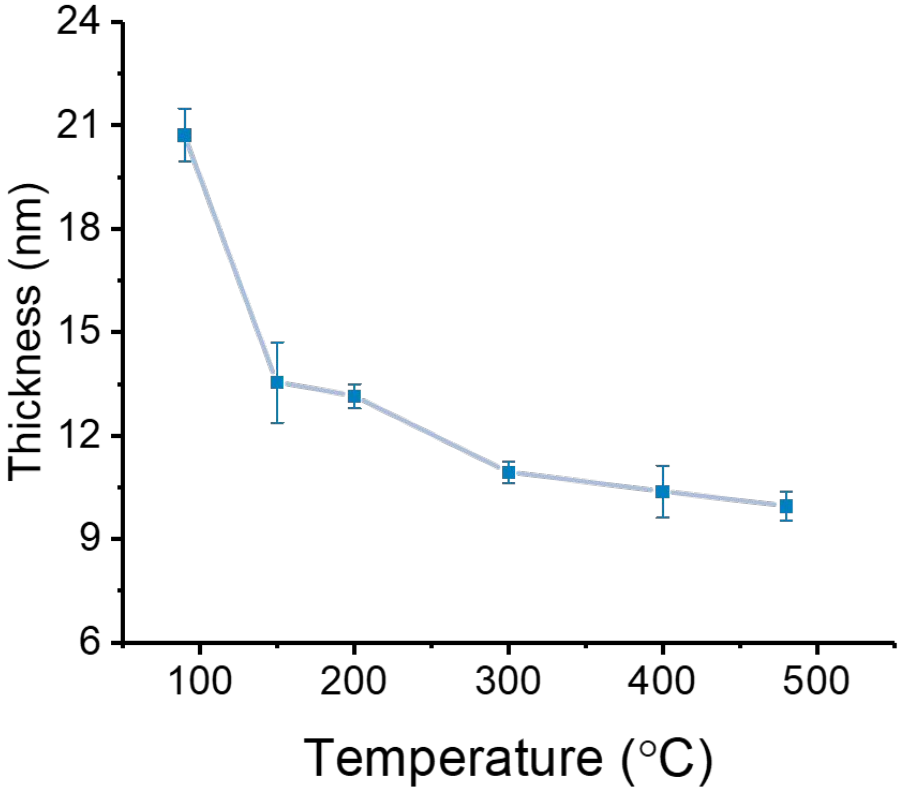

Figure S2. HfO_2 thicknesses in different temperatures on silicon wafers.

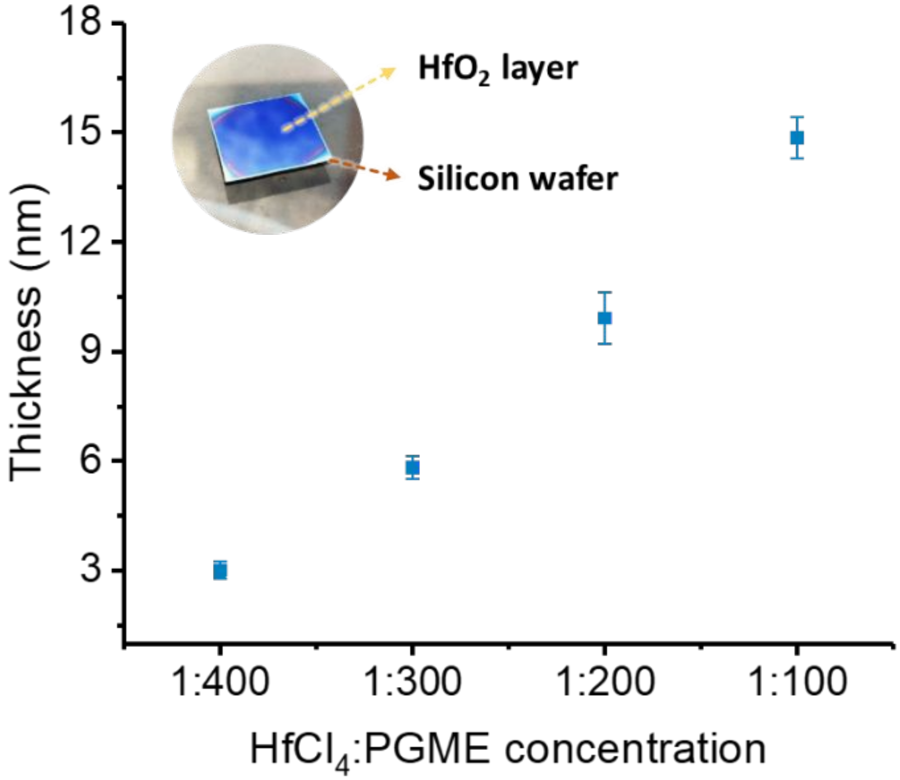

Figure S3. HfO_2 thicknesses at different molar ratios on silicon wafers.

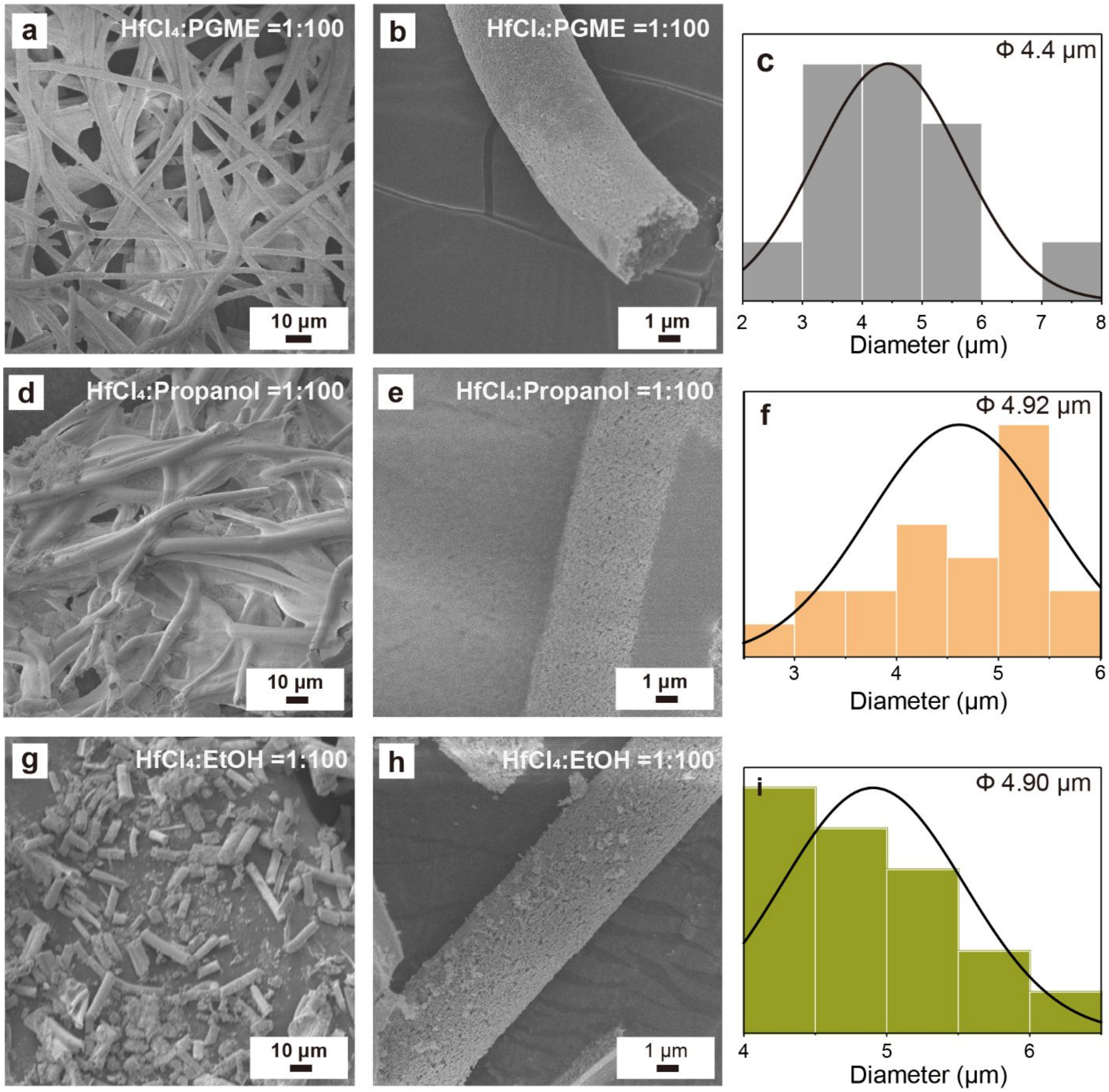

Figure S4. Hollow HfO_2 fibers prepared with different solvents. (a-c) PGME: (a,b) SEM images and (c) fiber diameter distribution. (d-f) Propanol: (d,e) SEM images and (f) fiber diameter distribution. (g-i) Ethanol: (g,h) SEM images and (i) fiber diameter distribution.

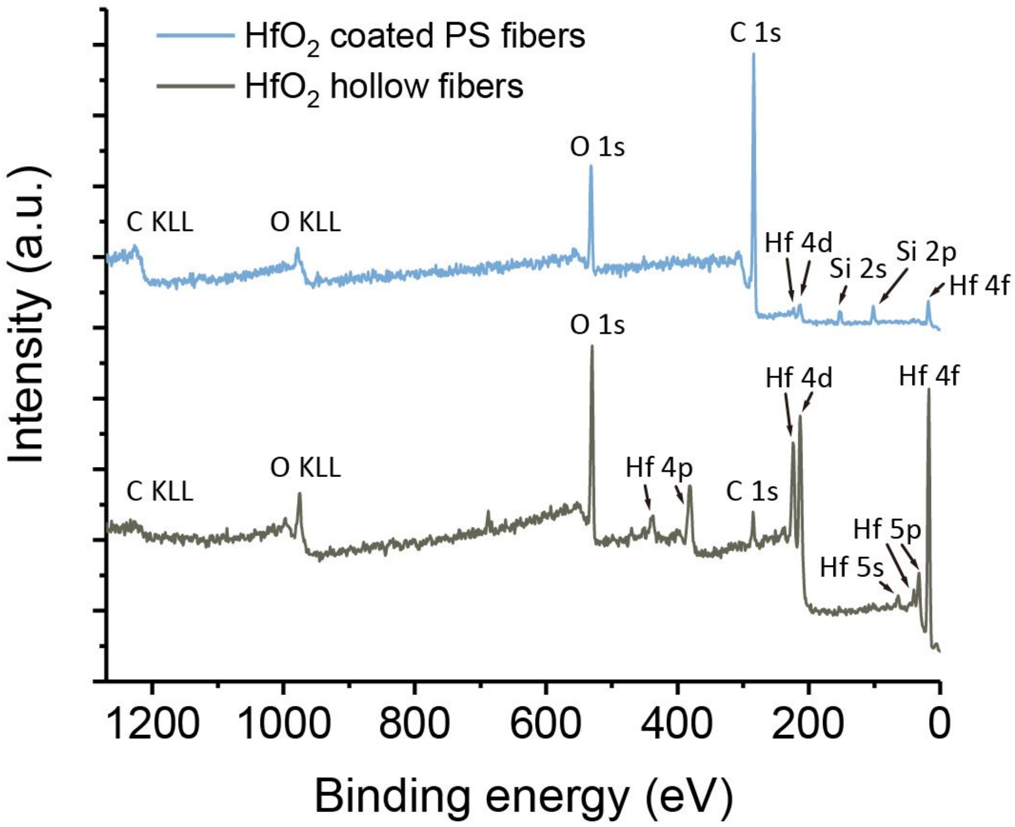

Figure S5. XPS data of the HfO_2 coated PS fibers and HfO_2 hollow fibers.
